# Impact of time-of-day and timing interval of neoadjuvant immunotherapy and chemotherapy infusions among patients with operable NSCLC using the inverse probability of treatment weighting method: a dual-center, retrospective study

**DOI:** 10.3389/fimmu.2026.1895311

**Published:** 2026-08-28

**Authors:** Xiang-yang Yu, Wen-yu Zhai, Zheng-zheng Xia, Kai Ma, Bai-hua Zhang, Xin Yu, Sheng-cheng Lin, Xiao-tong Guo, Ze-rui Zhao, Zhen-tao Yu

**Affiliations:** 1 Department of Thoracic Surgery, National Cancer Center/National Clinical Research Center for Cancer, Cancer Hospital and Shenzhen Hospital, Chinese Academy of Medical Sciences and Peking Union Medical College, Shenzhen, China; 2 Department of Thoracic Surgery, Sun Yat-sen University Cancer Center, State Key Laboratory of Oncology in South China, Guangdong Provincial Clinical Research Center for Cancer, Guangzhou, China; 3 Department of Pharmacy, National Cancer Center/National Clinical Research Center for Cancer, Cancer Hospital and Shenzhen Hospital, Chinese Academy of Medical Sciences and Peking Union Medical College, Shenzhen, China

**Keywords:** neoadjuvant immunotherapy, non-small cell lung cancer, prognosis, time-of-day, timing interval

## Abstract

**Background:**

Although multiple studies have confirmed that adjusting the time-of-day (ToDA) of immunotherapy and timing interval (TI) of chemoimmunotherapy can improve long-term outcomes in patients with advanced non-small cell lung cancer (NSCLC), whether this model similarly improves survival among patients with operable NSCLC has not been reported.

**Patients and methods:**

This dual-center, retrospective study included adult patients who received neoadjuvant chemoimmunotherapy (neoCIT) for clinical stage T1-4N0-3M0 NSCLC between January 2019 and December 2024. The impact of the ToDA of neoadjuvant immunotherapy (neoIO) on the efficacy and safety were analyzed. In addition, the inverse probability of treatment weighting (IPTW)-weighted Cox regression model was used to compare disease-free survival (DFS) and overall survival (OS) between patients who received either ≥75% (≥75% group) or <75% (<75% group) of the neoIO administrations before 14:00 h.

**Results:**

A total of 168 consecutive patients with a median follow-up of 30.3 months were included, of whom 123 patients (73.2%) were in the ≥75% group. Neoadjuvant treatment-related adverse events (neoTRAEs) occurred in 47.6% of the patients, including 63 (51.2%) and 17 (37.8%) patients in the ≥75% and <75% groups, respectively (*P* = 0.122). Pathologic complete response occurred in 31.7% of the patients in the ≥75% group and in 20.0% of those in the <75% group (*P* = 0.177); major pathologic response occurred in 59.3% and 48.9%, respectively (*P* = 0.300). According to the results of the IPTW-weighted univariate Cox analysis, patients in the <75% group had worse 3-year rates of DFS (77.1% vs. 77.9%; *P* = 0.065) and OS (68.3% vs. 89.1%; *P* = 0.033). These findings remained robust to multivariate Cox regression models (DFS: hazard ratio [HR]ffoo, 2.858; 95% confidence intervals [CIs]: 1.239-6.692; *P* = 0.014; and OS: HR, 7.625; 95% CIs: 1.112-52.272; *P* = 0.039). Additionally, a shorter mean TI of patients who received neoCIT infusions (<23.5 vs. ≥23.5 hours) emerged as an independent factor associated with better OS (HR, 0.143; 95% CIs, 0.024-0.875; *P* = 0.035).

**Conclusions:**

Although early infusions of most neoIO drugs did not affect safety or efficacy, it showed a potential trend toward improving long-term survival. The corresponding immune microenvironment and molecular mechanisms need further exploration to guide the design of future prospective clinical studies.

## 1 Introduction

The circadian rhythm affects immune functions by regulating the time-of-day (ToDA)-dependent production and transportation of cytokines/chemokines, as well as immune cell proliferation and tissue/tumor microenvironment infiltration (1–3). Additionally, the expression of programmed death 1 (PD-1), the key targeted protein in the PD-1/PD-ligand 1 (PD-L1) pathway that mediates tumor immune escape, in immune cells exhibits clear diurnal oscillation (4). A growing body of preclinical and clinical studies have confirmed that leveraging the circadian rhythm of the immune system to adjust the ToDA of immune checkpoint inhibitor (ICI) administration in patients with solid tumors, including melanoma, upper gastrointestinal tumors, and renal, hepatocellular, and bladder carcinomas, could help reduce side effects and improve treatment efficacy (1–6). A recent large, international, multicenter, retrospective cohort study in patients with advanced non-small cell lung cancer (NSCLC) also confirmed that compared with afternoon administration, morning infusion of first-line immunotherapy significantly increased patients’ objective response rate (ORR), progression-free survival (PFS), and overall survival (OS) (5). Currently, on the basis of the findings of multiple phase 3 randomized controlled trials (RCTs), the standard neoadjuvant therapy paradigm for resectable NSCLC without common driver gene mutations has shifted from chemotherapy alone to the combination of ICIs and chemotherapy (7–10). However, whether optimizing the ToDA of ICI administration during the neoadjuvant phase according to circadian rhythm could similarly enhance short- and long-term outcomes still lacks clinical evidence.

Although the National Comprehensive Cancer Network (NCCN) guidelines recommend administering neoadjuvant ICIs and chemotherapy to drive-negative resectable NSCLC patients on the same day, findings from both preclinical and clinical studies suggest that this may not represent the optimal timing interval (TI) strategy (6, 10, 11). Recent ex vivo and metastatic lung cancer mouse model studies have indicated that extending the TI (approximately 48 hours) between a PD-1 inhibitor and chemotherapy may enhance the antitumor immune response and considerably suppress tumor development, and these findings were further validated in a prospective clinical cohort of 170 patients with advanced NSCLC (6). Furthermore, another integrated study demonstrated that, compared with the concurrent combination, spacing out immunotherapy by 1 to 10 days after chemotherapy significantly improved OS in patients with refractory lung cancer (11). Therefore, exploring the optimum TI of preoperative ICIs and chemotherapy may be beneficial for improving the response and survival outcomes of patients with resectable NSCLC.

Here, leveraging a double-center, real-world cohort, we aimed to explore the effects of ToDA and TI of neoadjuvant chemoimmunotherapy (neoCIT) administrations on the efficacy and survival outcomes of patients with operable NSCLC.

This case-control study has been reported in line with the strengthening the report of cohort studies in surgery (STROCSS) 2024 guidelines (**Supplementary Table 1**) (12).

## 2 Patients and methods

### 2.1 Patient selection

**Figure 1:**
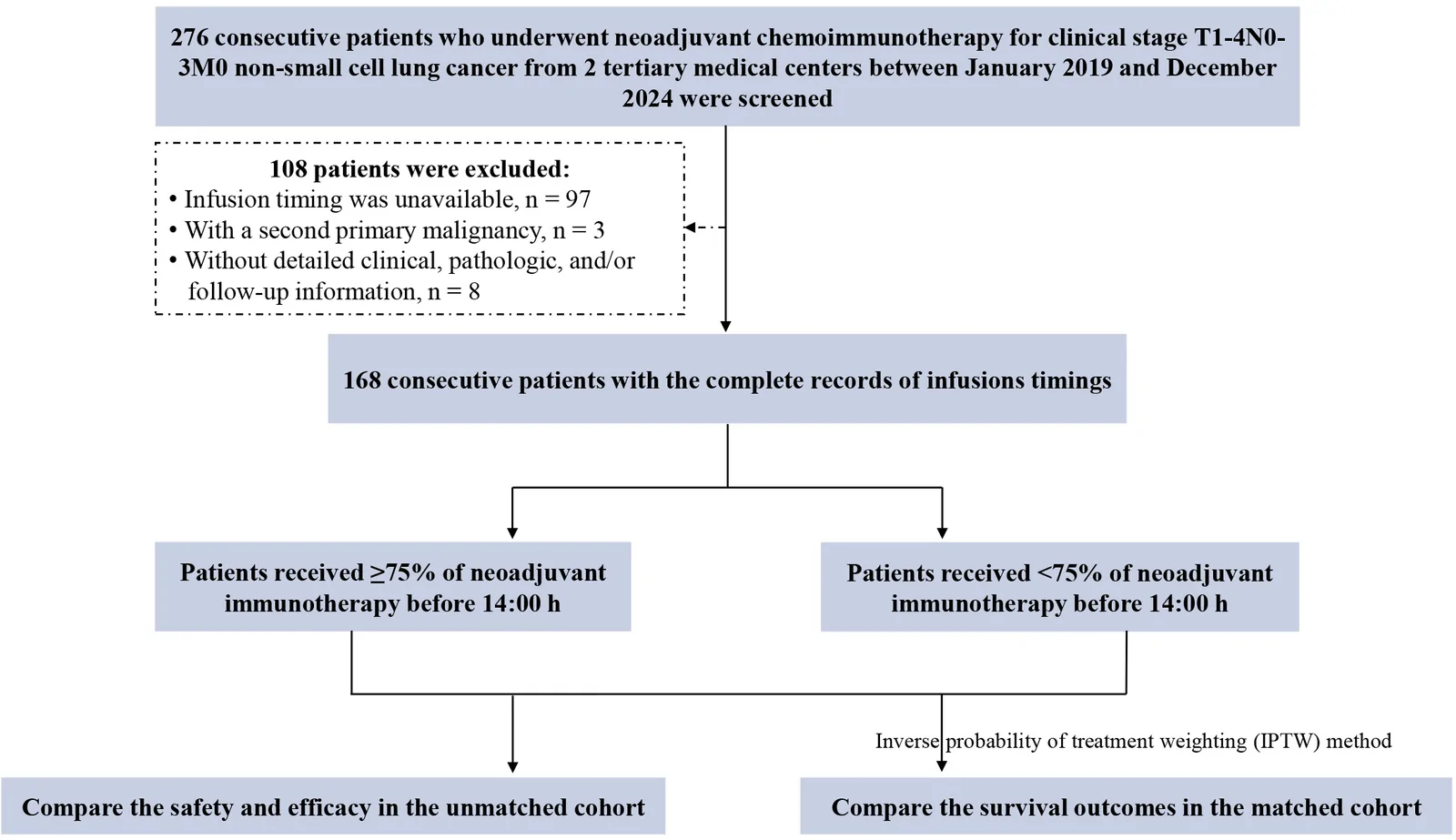
Study flowchart.

Patients who underwent surgery after chemoimmunotherapy for NSCLC between January 2019 and December 2024 were selected from 2 tertiary medical centres in southern China. The main eligibility criteria were as follows: i) based on the 9th edition of the TNM classification of lung cancer, the patient was staged as cT1-4N0-3M0; ii) radical resection was deemed achievable through local multidisciplinary team (MDT) discussion; and iii) age at first diagnosis was ≥18 years. Patients who lacked complete dates and timings of neoCIT infusions; who received additional neoadjuvant radiotherapy; who were diagnosed with other previous or subsequent primary invasive malignancies; who did not have detailed clinical, pathologic, or follow-up information; or who died within 30 days after surgery were excluded. The flowchart in **Figure 1** illustrates the patient recruitment process.

### 2.2 Neoadjuvant therapy and surgical procedures

Neoadjuvant ICIs (i.e., PD-1 or PD-L1 inhibitors) in combination with platinum-based doublet chemotherapy regimens were formulated by the local MDT on the basis of the NCCN and Chinese Society of Clinical Oncology guidelines for lung cancer, or prospective clinical trial protocols approved by the Institutional Review Board (8, 10, 13). Although the number of neoCIT cycles was primarily determined with reference to the aforementioned criteria, patient willingness, therapeutic efficacy, and toxic effects during the neoadjuvant phase were also important considerations. The exact records of dates and starting/ending times (hour:minute) of ICI and chemotherapy infusions were extracted from the nursing records. In the two participating centers, the routine anti-tumor drug infusions were administered daily from 9:00 h to 19:00 h. Therefore, the 14:00 h, as the midpoint, was selected as the dividing time point. The proportion of ICI infusions completed before 14:00 h was defined as the ratio of the total infusions times of neoadjuvant immunotherapy (neoIO) drugs administered before 14:00 h per cycle to the total infusion times of neoIO drugs per cycle. We used quartiles to group all patients. The 75% threshold was selected as the stratification cutoff primarily to align with findings from relevant preclinical and clinical studies (1–5, 14), maintain consistency with the stratification approaches used in published research (5, 14), and facilitate the clinical interpretation of the results. Mean TI was calculated as the sum of the TIs between neoadjuvant ICI and chemotherapy infusion per cycle, divided by the number of neoCIT cycles. The best cut-off values of mean TI were determined by maximum Youden index, which were generated by respective receiver operating characteristics (ROC) curves, to evaluate the predictive values in overall survival (OS, calculated from the initiation of the first cycle of neoCIT until the date of death from any cause).

The Common Terminology Criteria for Adverse Events (CTCAE, version 5.0) was utilized for recording and grading AEs during the neoCIT period. Surgical resection was generally scheduled at 28 to 42 days after completion of the last cycles of neoCIT. The surgical approach, either thoracotomy or minimally invasive surgery, was deliberated and agreed upon by the panel of thoracic surgery specialists at each center. The extent of resection included the primary tumor, along with the ipsilateral hilar and mediastinal lymph nodes. However, for patients with concomitant baseline N3 lymph node metastasis from our two investigator-initiated trials (ChiCTR2000040673 and ChiCTR2400081493), additional dissection was also needed. Surgical complications were defined and graded via the Clavien-Dindo classification.

### 2.3 Baseline and efficacy evaluations, follow-up, and outcomes

Magnetic resonance imaging (MRI) or computed tomography (CT) of the brain, contrast-enhanced CT of the neck, thorax and abdomen, and radionuclide whole-body bone scanning were carried out as routine baseline staging examinations. For N2 or N3 lymph nodes suspected of metastasis, invasive staging procedures (such as endobronchial ultrasound-guided transbronchial needle aspiration, endoscopic ultrasound-guided biopsy, video-assisted thoracoscopic surgery biopsy, mediastinoscopy, or ultrasound-guided fine-needle aspiration) and/or a positron emission tomography CT (PET/CT) scan (a maximum standardized uptake value ≥3 on PET and a short-axis diameter ≥10 mm on CT) were needed. Chest CT with contrast was performed within 7 days prior to surgery, and at least two experienced radiologists evaluated the radiographic response status according to the Response Evaluation Criteria in Solid Tumours (RECIST, version 1.1).

Pathologic assessment of the surgical specimens was performed by local seasoned pathologists as per the immune-related pathologic response criteria (irPRC). Pathologic complete response (pCR) was defined as the absence of any residual tumor cells observed in both the tumor bed and all the resected lymph nodes, whereas major pathologic response (MPR) was defined as a response in which only the proportion of residual tumor cells in the tumor bed did not exceed 10%.

Routine follow-up surveillance included chest CT and ultrasound examinations of the scalene and supraclavicular lymph nodes every 3 months for 2 years and then every 6 months for 3–5 years, followed by yearly follow-up. Consistent with the prospective RCTs and retrospective studies (9, 15, 16), disease-free survival (DFS) was defined as the time interval from the date of surgery to either lung cancer recurrence or death from any cause.

### 2.4 Statistical analysis

Continuous variables are presented as the mean (± standard deviation, SD) or median (interquartile range, IQR), and their differences were analyzed using Student’s t test or the Mann-Whitney U test, respectively. Differences between categorical variables, which are presented as numbers and percentages, were compared using the chi-square test or Fisher’s exact test. Our study employed a doubly robust estimation method that incorporates both inverse probability of treatment weighting (IPTW) and Cox regression adjustment to improve the robustness of survival analysis results. The IPTW method used the standardized mean difference (SMD) to balance baseline variables between the two groups of patients who received either ≥75% or <75% neoIO drugs before 14:00 h, where an SMD value less than 0.10 denoted good equilibrium (**Table 1**; **Supplementary Figure 1**). In the IPTW-weighted cohort, univariate Cox proportional hazards model was used to calculate the hazard ratios (HRs) and 95% confidence interval (CI), with accompanying survival curves generated using the Kaplan-Meier method. Variables with a *P* value <0.10 in the above univariate analysis were then included in the multivariate proportional hazards model to identify variables significantly associated with prognosis, defined as a two-sided *P* value <0.05. In addition, Firth’s penalized Cox regression model was employed to validate the trend of covariates in predicting survival. All the statistical analyses were conducted using R version 4.3.1 software.

**Table 1:** Clinical, radiologic, surgical and pathologic characteristics according to ≥75% (≥75% group) and <75% (<75% group) of patients who received neoadjuvant immunotherapy before 14:00 h.

Variables	Original cohort				Inverse probability of treatment weighting cohort			
	≥75% group (N = 123)	<75% group (N = 45)	SMD	*P* value	≥75% group (N = 166.6)	<75% group (N = 172.4)	SMD	*P* value
Age (years old), median (IQR)	64.0 (59.0-70.0)	64.0 (56.5-69.0)	0.051	0.893	64.0 (59.0-69.0)	63.3 (58.5-68.0)	0.018	0.923
Female sex, n (%)	15 (12.2%)	8 (17.8%)	0.157	0.497	21.4 (14.3%)	24.6 (14.3%)	0.041	0.834
Current/former smoker, n (%)	78 (63.4%)	32 (71.1%)	0.165	0.456	109.5 (65.8%)	106.7 (61.9%)	0.081	0.720
Histology, n (%)								
Squamous cell carcinoma	72 (58.5%)	25 (55.6%)	0.330	0.257	97.2 (58.4%)	105.6 (61.3%)	0.060	0.989
Adenocarcinoma	41 (33.3%)	16 (35.6%)			56.1 (33.7%)	54.2 (31.5%)		
LELC	8 (6.5%)	1 (2.2%)			9.2 (5.5%)	8.5 (4.9%)		
Others	2 (1.6%)	3 (6.7%)			4.0 (2.4%)	4.0 (2.3%)		
PD-L1 expression, n (%)								
Unknown	59 (48.0%)	21 (46.7%)	0.245	0.390	77.0 (46.2%)	68.2 (39.5%)	0.136	0.833
<1%	38 (30.9%)	18 (40.0%)			57.3 (34.4%)	66.6 (38.6%)		
≥1%	26 (21.2%)	6 (13.3%)			32.3 (19.4%)	37.7 (21.8%)		
Baseline tumor size, cm, mean (SD)	5.5 (1.9)	5.1 (2.2)	0.184	0.276	5.35 (1.86)	5.22 (1.84)	0.071	0.694
Clinical TNM staging, n (%)								
cI	2 (1.6%)	1 (2.2%)	0.045	0.965	2.8 (1.7%)	2.3 (1.3%)	0.159	0.706
cII	17 (13.8%)	6 (13.3%)			21.7 (13.0%)	32.4 (18.8%)		
cIII	104 (84.6%)	38 (84.4%)			142.1 (85.3%)	137.7 (79.9%)		
Neoadjuvant PD-1 inhibitor, n (%)	120 (97.6%)	44 (97.8%)	0.014	0.935	162.7 (97.7%)	169.7 (98.4%)	0.052	0.747
Neoadjuvant cycles, n (%)								
1-2	69 (56.1%)	27 (60.0%)	0.079	0.782	93.6 (56.2%)	87.4 (50.7%)	0.111	0.620
≥3	54 (43.9%)	18 (40.0%)			73.0 (43.8%)	85.1 (49.3%)		
Mean timing interval, n (%)								
<23.5 hours	10 (8.1%)	3 (6.7%)	0.056	0.753	12.2 (7.3%)	6.8 (4.0%)	0.146	0.347
≥23.5 hours	113 (91.9%)	42 (93.3%)			154.4 (92.7%)	165.6 (96.0%)		
Objective response, n (%)	83 (67.5%)	27 (60.0%)	0.156	0.472	111.7 (67.1%)	111.4 (64.6%)	0.053	0.810
Pathologic TNM staging, n (%)								
p0	39 (31.7%)	9 (20.0%)	0.350	0.258	47.9 (28.8%)	56.7 (32.9%)	0.098	0.966
pI	40 (32.5%)	16 (35.6%)			56.4 (33.9%)	56.0 (32.5%)		
pII	19 (15.4%)	12 (26.7%)			29.9 (17.9%)	26.8 (15.5%)		
pIII	25 (20.3%)	8 (17.8%)			32.3 (19.4%)	33.0 (19.1%)		
Major pathologic response, n (%)	73 (59.3%)	22 (48.9%)	0.211	0.300	94.2 (56.6%)	105.3 (61.1%)	0.091	0.664
Extent of resection, n (%)								
Sublobar resection	3 (2.4%)	0 (0%)	0.417	0.115	3.0 (1.8%)	0 (0%)	0.205	0.567
Lobectomy	105 (85.4%)	34 (75.6%)			139.3 (83.6%)	150.3 (87.2%)		
Bilobectomy	8 (6.5%)	8 (17.8%)			14.7 (8.8%)	14.7 (8.5%)		
Pneumonectomy	7 (5.7%)	3 (6.7%)			9.6 (5.8%)	7.5 (4.3%)		
Adjuvant therapy, n (%)	100 (81.3%)	36 (80.0%)	0.033	0.849	134.2 (80.6%)	142.2 (82.5%)	0.049	0.812

SMD, standardized mean difference; IQR, interquartile range; LELC, lymphoepithelioma-like carcinoma; PD-L1, programmed death ligand 1; SD, standard deviation; PD-1, programmed death 1.

## 3 Results

### 3.1 Patient characteristics

In this study, the 276 consecutive patients who underwent surgery after neoCIT were selected from a total of 337 consecutive patients who received initial neoCIT. Among them, 61 patients (18.1%) did not undergo subsequent surgery, primarily due to patient refusal of the recommended surgery (N = 29, 8.6%), followed by potential R1/R2 resection (N = 11, 3.3%), comorbidities rendering patients intolerant to surgery (N = 10, 3.0%), neoadjuvant treatment-related adverse events (neoTRAEs) (N = 9, 2.7%), and achieving radiographic complete response (N = 2, 0.6%). A total of 168 out of 276 consecutive patients (60.9%) met the selection criteria and were included in the final analysis (**Figure 1**).

The included patients received neoCIT mainly for clinical stage III (84.5%) NSCLC, including 97 (57.7%) with squamous cell carcinoma (SCC), 56 (33.9%) with adenocarcinoma, and 9 (5.4%) with lymphoepithelioma-like carcinoma. The majority were male (86.3%), with a median age of 64.0 (IQR, 58.0-69.0) years, and had a history of smoking (65.5%). More than half (52.4%) of the patients underwent PD-L1 testing via immunohistochemistry, of whom 32 had a PD-L1 tumor proportion score (TPS) of ≥1%. 164 patients (97.6%) received neoadjuvant PD-1 inhibitor therapy. Patients who received ≥75% of the neoIO administrations before 14:00 h were more likely to be male and nonsmokers, to have SCC, to test positive (≥1%) for PD-L1 expression, to have a smaller baseline tumor size, to have a lower pathologic stage, to undergo lobectomy than bilobectomy or pneumonectomy, and to achieve radiologic objective response and MPR (SMD ≥0.10 for all). All the aforementioned variables achieved good balance through the IPTW method (**Table 1**; **Supplementary Figure 1**).

### 3.2 neoTRAEs and surgical complications

During the neoadjuvant phase, TRAEs occurred in 47.6% of the patients, including 63 (51.2%) and 17 (37.8%) patients in the ≥75% (≥75% group) and <75% (<75% group) of the neoIO administrations before 14:00 h groups, respectively (**Supplementary Table 2**). A decreased neutrophil count (21.4%), increased alanine aminotransferase level (10.7%), alopecia (9.5%), and an increased aspartate aminotransferase level (6.5%) were the most common neoTRAEs; however, no neoadjuvant treatment-related death was recorded. Notably, three (1.8%) patients developed immune-related pneumonia, which was grade 1–2 in severity in all of the patients and did not require dose reduction or discontinuation of immunotherapy after steroid therapy. Neutropenia was the most common grade 3–4 neoTRAEs, with an incidence of 9.5%.

Postoperative pneumonia treated with broad-spectrum antibiotics on the ward, which was the most common surgical complication, occurred in 13 (7.7%) patients, among whom one developed lung failure and required intubation. Other relatively common surgical complications included prolonged air leakage (3.6%), arrhythmia (3.0%), blood transfusions (2.4%), second thoracic drainage (1.8%), and chylothorax (1.2%). The surgical complications were predominantly grade I-II, and none resulted in patient mortality during the perioperative period.

The incidence rates of each type of neoTRAE and surgical complication did not significantly differ between the ≥75% and <75% groups, as shown in **Supplementary Tables 2**, **3**.

### 3.3 Radiographic and pathologic assessments

**Figure 2:**
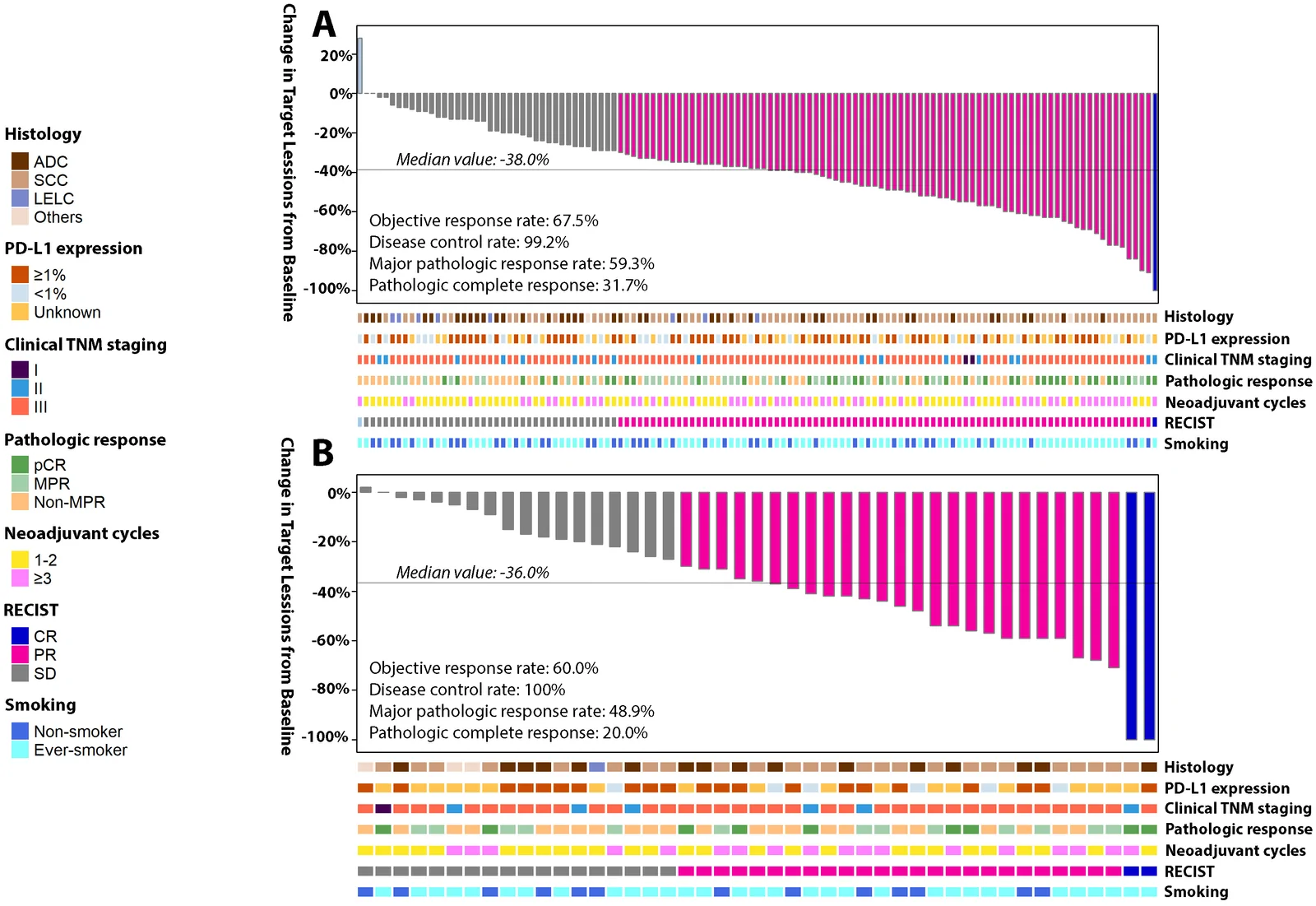
Radiographic evaluations and pathologic responses according to ≥75% **(A)** and <75% **(B)** of neoadjuvant immunotherapy received before 14:00 h. ADC, adenocarcinoma; SCC, squamous cell carcinoma; LELC, lymphoepithelioma-like carcinoma; PD-L1, programmed death ligand 1; pCR, pathologic complete response; MPR, major pathologic response; RECIST, response evaluation criteria in solid tumors.

The ORR was 67.5% (83/123; 95% CI: 58.8-75.1%) in the ≥75% group, with a median 38.0% decrease in the target lesion compared with baseline, and 60.0% (27/45; 95% CI: 45.5-73.0%) in the <75% group, with a median 36.0% decrease (**Table 1**; **Figure 2**; *P* = 0.472).

100 patients (59.5%) underwent minimally invasive surgery, and most patients underwent lobectomy (139/168, 82.7%) (**Supplementary Table 2**). No significant differences were detected between the ≥75% and <75% groups in terms of operative time, estimated blood loss, or length of postoperative hospital stay (all *P*>0.05). After surgical resection, MPR occurred in 73 patients (59.3%, 95% CI: 50.5-67.6%) in the ≥75% group compared with 22 patients in the <75% group (48.9%, 95% CI: 35.0-63.0%; *P* = 0.300) (**Figure 2**).

### 3.4 Survival outcomes

**Figure 3:**
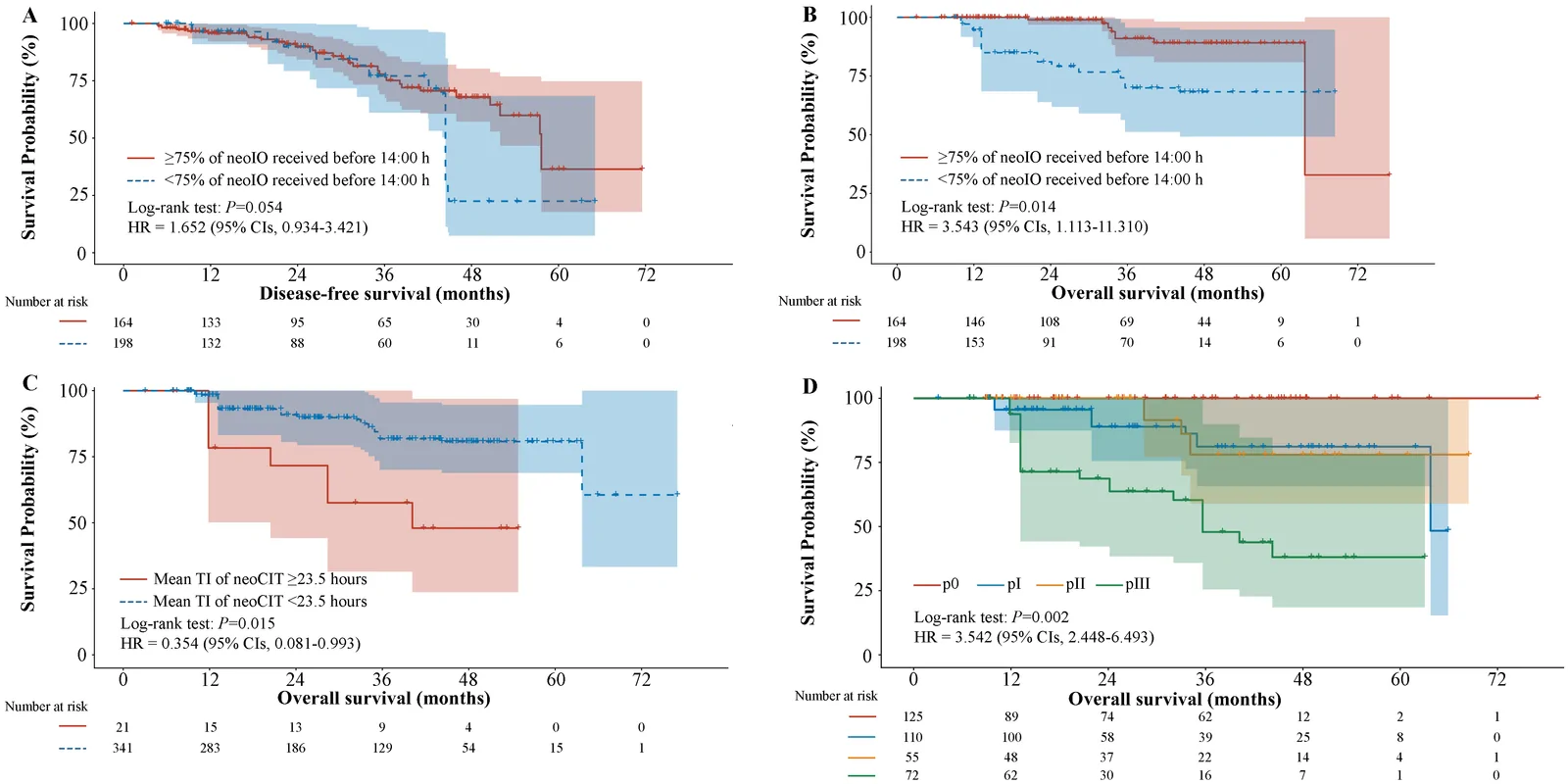
Inverse probability of treatment weighting (IPTW)-weighted Kaplan-Meier analysis of disease-free survival [DFS, **(A)**] and overall survival [OS, **(B)**] in patients who received ≥75% and <75% neoadjuvant immunotherapy (neoIO) before 14:00 h. IPTW-weighted Kaplan-Meier analysis of OS according to the mean timing interval of neoadjuvant chemoimmunotherapy [neoCIT, **(C)**] and pathologic TNM stage **(D)**.

During a median follow-up of 30.3 months (IQR, 17.3-45.7 months, as of August 31, 2025) in the real-world cohort, 43 patients (25.6%) developed recurrence and 16 (9.5%) died, with 13 (7.7%) died of lung cancer. The estimated 3-year DFS and OS rates were 75.7% (95% CIs: 71.5-79.9%) and 87.0% (95% CIs: 83.5-90.5%), respectively. In the IPTW-weighted cohort, univariate analysis demonstrated that patients with <75% of neoIO infusions administered before 14:00 h had significantly shorter DFS (median 44.4 vs.57.6 months; 3-year rate: 77.1% vs.77.9%; *P* = 0.065) (**Figure 3A**) and OS (median NR vs.63.7 months; 3-year OS: 68.3% vs. 89.1%; *P* = 0.033) (**Figure 3B**) compared to those in the ≥75% group. The adverse impact on survival remained statistically significant in the multivariate analyses (DFS: HR, 2.858, 95% CIs: 1.239-6.692, *P* = 0.014; and OS: HR, 7.625, 95% CIs: 1.112-52.272, *P* = 0.039) (**Table 2**). Additionally, the Cox proportional hazards model also identified the PD-L1 expression status and the extent of resection as the independently significant prognostic factors for DFS. Factors associated with significantly prolonged OS also included the shorter mean interval of neoCIT infusions (<23.5 hours compared with ≥23.5 hours) (**Figure 3C**) and earlier pathological TNM staging (**Figure 3D**). Firth’s penalized Cox regression analyses (**Supplementary Table 4**) consistently showed that, compared with patients in the ≥75% group, those in the <75% group had worse DFS (HR, 2.141, 95% CIs: 0.923-4.969, *P* = 0.076) and OS (HR, 3.295, 95% CIs: 0.831-13.072, *P* = 0.090). In addition, if patients received neoCIT with mean TI <23.5 hours, it led to better OS (HR, 0.063, 95% CIs: 0.007-0.533, *P* = 0.011).

**Table 2:** Univariable and multivariable Cox proportional hazards models for variables associated with disease-free survival and overall survival in the inverse probability of treatment weighting cohort.

Variables	Disease-free survival				Overall survival			
	Univariable		Multivariable		Univariable		Multivariable	
	HR (95% CIs)	*P* value	HR (95% CIs)	*P* value	HR (95% CIs)	*P* value	HR (95% CIs)	*P* value
Age								
<60 years old	Reference				Reference		Reference	
≥60 years old	1.605 (0.770-3.344)	0.191			3.950 (0.966-16.160)	0.054	4.031 (0.965-16.830)	0.056
Sex								
Male	Reference				Reference			
Female	0.604 (0.288-1.267)	0.213			0.601 (0.226-1.595)	0.443		
Smoking history								
Current/former	Reference		Reference	0.396	Reference			
Never	0.435 (0.236-0.803)	0.004	0.685 (0.285-1.643)		0.380 (0.161-0.901)	0.152		
Histology, n (%)								
Squamous cell carcinoma	Reference				Reference			
Adenocarcinoma	1.336 (0.686-2.602)	0.395			1.231 (0.307-4.941)	0.769		
LELC	0.867 (0.279-2.700)	0.806			0.412 (0.093-0.827)	<0.001		
Others	4.160 (1.580-10.951)	0.004			1.290 (0.180-9.224)	0.800		
PD-L1 expression								
Unknown	Reference		Reference		Reference			
≥1%	1.876 (0.992-3.816)	0.082	2.784 (1.203-6.444)	0.017	2.999 (0.428-21.030)	0.269		
<1%	3.003 (1.211-7.449)	0.018	4.469 (1.725-11.576)	0.002	4.980 (1.000-24.790)	0.050		
Baseline tumor size								
<5 cm	Reference				Reference			
≥5 cm	1.061 (0.578-1.946)	0.844			1.805 (0.760-4.288)	0.335		
Neoadjuvant ICIs								
PD-1 inhibitor	Reference				Reference			
PD-L1 inhibitor	0.259 (0.015-4.432)	0.247			0.395 (0.105-1.497)	0.140		
Neoadjuvant cycles								
1-2	Reference				Reference		Reference	
≥3	0.939 (0.516-1.708)	0.833			0.305 (0.113-0.828)	0.084	2.525 (0.433-14.734)	0.303
ToDA of neoIO								
≥75% before 14:00 h	Reference		Reference		Reference		Reference	
<75% before 14:00 h	1.742 (0.946-3.210)	0.065	2.858 (1.239-6.592)	0.014	3.544 (1.340-9.369)	0.033	7.625 (1.112-52.272)	0.039
Mean timing interval								
≥23.5 hours	Reference		Reference		Reference		Reference	
<23.5 hours	0.326 (0.143-0.741)	0.020	0.408 (0.100-1.663)	0.211	0.274 (0.095-0.789)	0.048	0.143 (0.024-0.875)	0.035
Radiographic response								
PR + CR	Reference		Reference		Reference			
SD + PD	2.142 (1.162-3.950)	0.010	1.825 (0.811-4.108)	0.146	0.308 (0.101-0.945)	0.122		
Pathologic TNM staging								
p0	Reference				Reference		Reference	
pI	0.692 (0.252-1.901)	0.476			2.368 (1.023-6.493)	<0.001	2.509 (1.113-5.379)	<0.001
pII	0.757 (0.245-2.333)	0.628			4.197 (2.450-6.220)	<0.001	2.971 (1.149-6.227)	<0.001
pIII	3.339 (1.585-7.033)	0.002			8.010 (2.977-21.550)	<0.001	5.047 (2.894-7.054)	<0.001
Major pathologic response								
No	Reference				Reference		Reference	
Yes	0.695 (0.382-1.263)	0.313			0.118 (0.039-0.354)	0.003	0.197 (0.030-1.304)	0.092
Extent of resection								
Sublobar resection	Reference		Reference		Reference			
Lobectomy	0.123 (0.025-0.608)	0.010	0.107 (0.019-0.605)	0.011	0.212 (0.013-1.731)	0.985		
Bilobectomy	0.179 (0.031-1.008)	0.051	0.138 (0.021-0.901)	0.039	0.537 (0.070-4.137)	0.550		
Pneumonectomy	0.152 (0.016-1.429)	0.100	0.072 (0.003-1.748)	0.106	0.818 (0.072-9.277)	0.872		
Adjuvant therapy								
Yes	Reference				Reference			
No	1.693 (0.518-5.531)	0.338			2.082 (0.442-9.780)	0.381		

HR, hazard ratio; CIs, confidence intervals; LELC, lymphoepithelioma-like carcinoma; PD-L1, programmed death ligand 1; ICIs, immune checkpoint inhibitors; ToDA, time-of-day; neoIO, neoadjuvant immunotherapy; PD-1, programmed death 1; PR, partial response; CR, complete response; SD, stable disease; PD, progressive disease.

## 4 Discussion

To our knowledge, this is the first study investigating the infusion timing of neoIO in operable NSCLC, which demonstrated that patients who received ≥75% of neoIO infusions before 14:00 h had significantly longer survival times than those who received <75% of infusions before 14:00 h did, as evidenced by the IPTW-weighted Cox models. Additionally, we also observed that a shorter mean interval (<23.5 hours) of neoCIT infusions correlated with improved OS. Taken together, these findings strongly underscore the necessity of optimizing the timing, interval, and sequence of neoCIT administrations, as well as exploring the underlying mechanisms.

Previous preclinical studies in mammals revealed that tumor-infiltrating immune cells, which affect the proliferation, differentiation, and metastasis of tumor cells, are influenced by the circadian clock (around 24 hours) (1, 3, 4, 17). Additionally, multiple experiments further confirmed that compared with those in the “afternoon” or “evening” group, immunological T cells and gene clusters involved in T-cell activation pathways exhibited higher or peak expression in the “morning” group, which offers practical guidance for the ToDA of antitumor immunotherapy infusion (3, 18). Furthermore, research on the pharmacokinetics and biodistribution of radiolabeled pembrolizumab in mice and rats has shown that anti-PD-1 inhibitors are rapidly cleared by certain tissues within 12 hours after infusion (19). Therefore, it is imperative to optimize the ToDA of immunotherapy administration in clinical practice on the basis of circadian antitumor immune regulation and the mechanisms of ICIs metabolism. As early as 2022, Abdoulaye, K and colleagues from France investigated the ToDA of second- or later-line nivolumab monotherapy in patients with metastatic NSCLC. Their results indicated that receiving the majority of nivolumab infusions before 12:54 h was a significant predictor of longer progression-free survival (PFS) and OS (14). Subsequently, together with Chinese researchers, they retrospectively collected data from treatment-naïve, advanced NSCLC patients across two different continents and time zones. The results of the propensity score matching analysis and multivariate survival analysis confirmed that the administration of ICIs before 11:30 h was significantly correlated with a higher ORR and longer PFS and OS (5). Consistent with the aforementioned literature, we selected the midpoint (14:00 h) of the daily antineoplatic drugs infusions period as the cutoff point in the current retrospective study on neoIO for NSCLC (5, 14). This time point not only aligned with current clinical practice, but also facilitated clinicians in designing infusion schedules for patients. Similarly, our findings also supported this optimization, showing that administering most neoadjuvant immunotherapeutic agents earlier in the day (before 14:00 h) significantly reduced the risk of disease recurrence or death, and this knowledge provides a theoretical foundation for designing future prospective RCTs.

Our results showed that although adjusting the ToDA of ICIs infusion had a significant impact on survival outcomes, it did not affect neoTRAEs or surgical complications, which was largely consistent with the findings reported by David et al. in patients with advanced melanoma (20). However, Karaboué et al. reported a significantly higher incidence of grade 2–3 skin toxicities and lower grade 3–4 fatigue among patients with metastatic NSCLC who received the majority of their nivolumab infusions in the morning group compared to those who received them in the afternoon group (14). These observations highlight the necessity of prospective research exploring how circadian rhythms influence both the adverse effects and therapeutic outcomes of ICIs, as this aspect remains poorly understood and demands future investigation to optimize treatment regimens. In addition, a subject-based counting method was used to record each neoTRAEs and its highest grade, without accounting for the impact of dose adjustments on AEs and grades. As a result, the total number and overall intensity of neoTRAEs were not fully captured.

Several cellular and animal experiments revealed that the efficacy of anti-PD-1 or PD-L1 inhibitors in solid tumors was influenced by activated CD8^+^ T cells and high PD-L1 expression (6, 21, 22). Cytotoxic drugs, such as cisplatin and oxaliplatin, can induce tumor cell death and stimulate the release of tumor-related antigens, thereby activating tumor-specific CD8^+^ T cells and promoting PD-L1 expression (6). However, this effect peaked rapidly during the initial phase of chemotherapy infusions and subsequently diminished (23). Therefore, an important clinical issue is how to adjust the TI and sequence of immunotherapy and chemotherapy on the basis of the above mechanisms to maximize the antitumor benefits. In 2021, a retrospective study revealed that the use of PD-1 or PD-L1 antibodies 3–5 days after chemotherapy was superior to the use of PD-1 or PD-L1 antibodies before or concurrent with chemotherapy in 64 patients with refractory lung cancer (11). Recently, preclinical research from Yi Zhang and colleagues demonstrated that compared with that on day 1 and day 2 after chemotherapy, the PD-1 expression on CD8+ T-cell and T-cell function gradually improved on day 3, while PD-L1 expression on myeloid-derived suppressor cells decreased significantly (6). In their subsequent NSCLC patient cohort, compared with the concurrent chemoimmunotherapy group, the group receiving immunotherapy 3 days after chemotherapy had a higher ORR and disease control rate, and prolonged PFS (6). In this retrospective study, patients with operable NSCLC received neoCIT either on the same day or on alternating days, on the basis of protocols recommended by phase III RCTs and guidelines (7–10, 13). Our findings under the above premise suggested that a shorter TI (<23.5 hours) could lead to a more favorable prognosis, which was verified in a retrospective study of advanced esophageal carcinoma patients who received CIT on the same day (16). Owing to the small sample sizes and short follow-up times of the above studies and inconsistent findings in other solid tumors (15, 16), the available evidence remains insufficient to determine the optimal TI and sequence of immunotherapy and chemotherapy.

Apart from the obvious limitations, such as its retrospective nature, the recruitment of patients from a single time zone, and the lack of external validation, the following limitations should also be noted when the results of this study are interpreted. First, the ToDA and TI of the neoCIT administrations in this study may have been influenced by other nononcological confounding factors, such as the COVID-19 pandemic, working hours, and holidays. Second, PD-1 and PD-L1 inhibitors as were adjusted for in this study, the potential impact of different immunotherapeutic agents on the outcomes was not adequately explored. Third, whether different chemotherapy agents and doses affect the optimal ToDA for immunotherapy administrations remains unknown. Fourth, as a retrospective study, we could only identify and quantify the independent effects of multiple independent variables on the dependent variable under the premise of controlling for known and available confounders. Consequently, the findings may be challenged as additional independent variables are introduced. Fifth, the modest sample size and relatively low number of events inherently limited the statistical power of our multivariable analyses. This was evidenced by the sensitivity analysis using Firth’s penalized regression, which, while demonstrating directional consistency with the primary IPTW-weighted analysis, exhibited attenuated statistical significance for certain secondary covariates. This attenuation is consistent with the conservative nature of Firth’s model and underscores the exploratory nature of these secondary associations. Sixth, to address our research objectives, we established corresponding inclusion and exclusion criteria during the case screening process, which may potentially introduce selection bias. Finally, the optimal ToDA (14:00 h) for immunotherapy administrations in this study was determined on the basis of survival curves rather than on findings from basic or clinical research. Future prospective, international multi-center, RCTs across different ethnicities, time zones, and countries are needed to verify and ultimately elucidate the optimal ToDA, TI, and sequence of neoCIT for patients with operable NSCLC.

## 5 Conclusion

In conclusion, unlike the well-established, high-quality evidence regarding the optimal ToDA of first-line immunotherapy in advanced NSCLC, whether adjusting the ToDA of neoIO similarly improves survival in early-stage NSCLC has not been explored. On the basis of a multi-center, large-sample retrospective cohort, our study is the first to demonstrate that earlier neoIO administrations, along with a shorter TI of neoCIT on the same day or on alternating days, can improve patient OS without incurring additional costs.

## Data Availability

The original contributions presented in the study are included in the article/**Supplementary Material**. Further inquiries can be directed to the corresponding authors.
