# Supplementary material for: Impact of time-of-day and timing interval of neoadjuvant immunotherapy and chemotherapy infusions among patients with operable NSCLC using the inverse probability of treatment weighting method: a dual-center, retrospective study
Source: Front Immunol. 2026 Aug 28;17:1895311. doi: 10.3389/fimmu.2026.1895311 (PMC13561947; doi:10.3389/fimmu.2026.1895311)
Supplement: Supplementary file 1 [file Table1.docx]

Supplementary Table 1: The strengthening the report of cohort studies in surgery (STROCSS) 2024 guidelines checklist.

| **Topic** | **Item** | **Item description** | **Page number** |
| --- | --- | --- | --- |
| Title | 1 | *Title*  The word 'cohort' or 'cross-sectional' or 'case-control' is included*  Temporal design of the study is stated (e.g. retrospective or prospective)  The focus of the study is clearly stated (e.g. population, setting, disease, exposure/intervention, outcome, etc.)  **STROCSS 2024 guidelines apply to all observational studies (e.g. cohort, cross-sectional, case-control, etc.)* | Page 1 |
| Highlights | 2 | *Highlights*  Include three to five bullet points that summarise the key findings of the study.  Provide a brief background to the study, the key results and clinical relevance. | Pages 1-2 |
| Abstract | 3a | *Structure*  Provide a structured abstract that includes the following headings:  1. Background  2. Methods  3. Results  4. Conclusions | Pages 1-2 |
|  | 3b | *Background*  Briefly describe:  Relevant context  Scientific rationale for this study  Aims and objectives | Page 1 |
|  | 3c | *Methods*  Briefly describe:  Type of study design (e.g. cohort, case-control, cross-sectional etc.)  Specification of study design (e.g. retro-/prospective, single/multicentred etc.)  All patient groups involved, including control group, if applicable  Exposure/interventions (e.g. type, operators, recipients, dates and time frames etc.)  Outcome measures- explicitly state primary and secondary outcome(s), where appropriate  Statistical methods of assessment used, where applicable | Page 1 |
|  | 3d | *Results*  Briefly describe:  Summary data  Principal findings with qualitative descriptions  Statistical findings and their significance, where appropriate | Pages 1-2 |
|  | 3e | *Conclusion*  Describe key conclusions briefly  Refer to implications for clinical practice and public health  Describe the need for and direction of future research  Include a concise statement that encapsulates the significance of the research and its contribution to the field | Page 2 |
| Keywords | 4 | *Keywords*  Include three to six keywords that identify what is covered in the study (e.g. patient population, diagnosis, or surgical intervention)  Include study type as a keyword (e.g. cohort study, cross-sectional study, case-control study etc.)  Include surgical speciality as one of the keywords  Include study location as one of the keywords | Page 2 |
| Introduction | 5a | *Introduction*  By referencing key literature throughout, comprehensively describe:  Relevant background and scientific rationale for study  Aims and objectives  Research question and hypotheses, where appropriate  Potential impact of research on future clinical practice  Economic relevance of study to society | Page 2 |
|  | 5b | *Guideline citation*  At the end of the introduction, refer to the STROCSS 2024 publication by stating: ‘This cohort/cross- sectional/case-control study has been reported in line with the STROCSS guidelines [include citation]’ | Page 2 |
| Methods: Study design | 6a | *Study design*  State the type of study design (e.g. cohort, cross-sectional, case-control etc.)  Describe other key elements of study design (e.g. retro-/prospective, single/multi-centred etc.)  Specify the duration of the study, including start and end dates | Page 3 |
|  | 6b | *Setting and timeframe of research*  Comprehensively describe:  Specific geographical location  Nature of institution (e.g. primary/secondary/tertiary care setting, district general hospital/teaching hospital, public/private, low-resource setting etc.)  Timeline for study, including dates for recruitment, exposure, follow-up, data collection etc.  Any deviations from the initial study design plan or changes to the timeline during the research, with reasons and implications stated | Page 3 |
|  | 6c | *Study groups*  Total number of participants  Number of groups  Number of participants in each group  Detail exposure/intervention allocated to each group  Inclusion and exclusion criteria with clear definitions | Page 3 |
|  | 6d | *Subgroup analysis*  Comprehensively describe:  How subgroups were defined  Planned subgroup analyses  Methods used to examine subgroups and their interactions | Page 3 |
|  | 6e | *Follow-up*  If applicable, comprehensively describe:  Time, length, frequency, location and methods of follow-up (e.g. mail, telephone, with whom etc.)  Any specific long-term surveillance requirements (e.g. imaging surveillance of endovascular aneurysm repair)  Any specific postoperative instructions (e.g. postoperative medications, targeted physiotherapy etc.) | Pages 3-4 |
| Methods: Participant Recruitment | 7a | *Recruitment*  Comprehensively describe:  Period of recruitment  Methods of recruitment to each patient group (e.g. all at once, in batches, continuously till desired sample size is reached etc.)  Sources of recruitment (e.g. physician referral, study website, social media, posters etc.)  Any monetary/nonmonetary incentivisation of participants to encourage involvement should be declared (the nature of any incentives provided must be clarified)  Any challenges encountered during the recruitment processes, including how they were addressed | Page 3 |
|  | 7b | *Sample size*  Comprehensively describe:  Analysis to determine optimal sample size for study accounting for population/effect size  Power calculations with justifications for chosen statistical power, where appropriate  Margin of error calculation Any associated ethical considerations | Page 3 |
| Methods: Intervention and Outcomes | 8a | *Pre-intervention considerations*  Comprehensively describe any preoperative patient optimisation:  Lifestyle optimisation (e.g. weight loss, smoking cessation, glycaemic control etc.)  Medical optimisation (e.g. medication review, treating hypothermia/-volemia/-tension, ICU care etc.)  Procedural optimisation (e.g. nil by mouth, enema etc.)  Other (e.g. psychological support, physiotherapy etc.) | Page 3 |
|  | 8b | *Intervention*  Comprehensively describe:  Type of intervention and reasoning (e.g. pharmacological, surgical, physiotherapy, psychological etc.)  Aim of intervention (e.g. preventative/therapeutic)  Total cost of performing the intervention  Degree of novelty of intervention  Any learning required for intervention  Prevalence or frequency at which the intervention is performed  Concurrent treatments (e.g. antibiotics, analgesia, antiemetics, VTE prophylaxis etc.)  Manufacturer and model details, where appropriate | Page 3 |
|  | 8c | *Intraintervention considerations*  Using figures and other media to illustrate wherever appropriate, comprehensively describe:  Details pertaining to administration of intervention (e.g. anaesthetic, positioning, location, preparation, equipment needed, devices, sutures, operative techniques, operative time etc.)  For pharmacological therapies, the formulation, dosages, routes, strength and durations  For surgery, any postoperative instruction (e.g. when to remove staples or sutures)  The degree of novelty for a surgical technique/device (e.g. ‘first in human’) | Page 3 |
|  | 8d | *Operator details*  Comprehensively describe:  Requirement for additional training  Learning curve for technique, including how it was evaluated (e.g. number of cases required to reach a defined level of proficiency)  Relevant training, specialisation, and operator's experience (e.g. average number of the relevant procedures performed annually)  Any institutional support that was provided to operators to facilitate their training | Page 3 |
|  | 8e | *Setting of intervention*  Comprehensively describe:  Setting in which the intervention was performed  Level of experience the centre has in performing the intervention | Page 3 |
|  | 8f | *Quality control*  Comprehensively describe:  Measures taken to reduce interoperator variability (e.g. regular team meetings, calibration exercises)  Measures taken to ensure consistency in other aspects of intervention delivery (e.g. data collection)  Measures taken to ensure quality in intervention delivery | Page 4 |
|  | 8g | *Postintervention considerations*  Comprehensively describe:  Postoperative instructions and care (e.g. avoid heavy lifting, dietary restrictions etc.)  Follow-up measures  Future surveillance requirements (e.g. blood tests, imaging etc.)  How patient engagement with postintervention instructions will be encouraged and monitored  If applicable, the criteria for patient discharge from the medical facility | Pages 3-4 |
|  | 8h | *Definition of outcomes*  Define primary outcomes, including validation with full reference to relevant studies, where applicable  Define secondary outcomes, where appropriate  Describe methods or instruments used to measure each outcome, with full reference given if validated  Describe follow-up period for outcome assessment, divided by group | Page 3 |
|  | 8i | *Statistics*  Comprehensively describe:  Statistical tests and statistical package(s)/software used  Rationale behind the statistical tests/software of choice  Confounders and their control, if known  Analysis approach (e.g. intention to treat/per protocol)  Any subgroup analyses  Level of statistical significance  How the results of the statistical analyses are presented (e.g. *P* values, confidence intervals, point estimates etc.) | Page 4 |
| Results | 9a | *Participants*  Comprehensively describe:  With reasons, the flow of participants (recruitment, nonparticipation, cross-over and withdrawal), using a figure to illustrate where appropriate  Population demographics (e.g. age, gender, relevant socioeconomic features, prognostic features etc.)  Any significant numerical differences across groups  If applicable, the longitudinal changes in participant flow/demographics over time | Pages 4-6 |
|  | 9b | *Participant comparison*  Include table comparing baseline characteristics of cohort groups, with statistical data included  Concisely, highlight the principal, significant findings  Describe any group matching, with methods | Pages 4-5 |
|  | 9c | *Outcomes*  Comprehensively describe:  Clinician-assessed and patient-reported outcomes (e.g. questionnaires with quality-of-life scales) for each group  Expected versus attained outcomes, as assessed by the clinician*  Primary and secondary outcomes, as previously defined (Item 8h)  Details of when the outcomes were recorded (e.g. at how many months/years postoperatively)  Relevant photographs and imaging are desirable  Any confounding factors and state which ones are adjusted and how  Any changes to interventions, with rationale and diagram, if appropriate  **NB*: reference relevant literature to inform expected outcomes | Pages 4-6 |
|  | 9d | *Tolerance*  Comprehensively describe:  Assessment of tolerability of exposure/intervention within patient groups  Methods of measuring tolerance/adherence  If applicable, specific patient perspectives  Whether these results will have an impact on the long-term applicability of the findings in clinical practice  Loss to follow-up (fraction and percentage), with reasons | Pages 4-6 |
|  | 9e | *Complications*  Comprehensively describe:  Adverse events, classified according to the Clavien-Dindo classification*  Timing of adverse events  Precautionary measures taken to prevent complications (e.g. antibiotic or venous thromboembolism prophylaxis)  Management of adverse events (e.g. blood transfusion, wound care, revision surgery etc.)  If applicable, whether the complication was reported to the national agency/pharmaceutical company  If applicable, specify whether any complications were discussed locally and the impact of such discussions (e.g. during team morbidity & mortality meetings)  State explicitly if there were no complications/adverse outcomes  **Dindo D, Demartines N, Clavien P-A. Classification of Surgical Complications. A New Proposal with Evaluation in a Cohort of 6336 Patients and Results of a Survey. Ann Surg. 2002; 240(2): 205-213* | Page 5 |
|  | 9f | *Key results*  Describe:  Key findings, supported by relevant raw data and corresponding statistical analyses with significance | Pages 4-6 |
| Discussion | 10a | *Principal findings*  By referencing key, relevant literature throughout, comprehensively describe:  Summary of key findings and conclusions  Rationale behind conclusions drawn  Comparison to current gold standard of care, current guidelines or similar research  Implications of findings for future clinical practice and guidelines  Relevant hypothesis generation | Pages 6-8 |
|  | 10b | *Strengths and limitations*  Comprehensively describe:  Strengths of the study  Weaknesses and limitations of the study  Measures taken to overcome the limitations, if applicable  Potential impact on results and their interpretation  Assessment and management of bias  Deviations from protocol, with reasons stated | Pages 7-8 |
|  | 10c | *Relevance and implications*  Comprehensively describe:  Relevance of findings  Potential implications for future clinical practice and guidelines  Measures that can be taken to enhance the quality of research study  Need for and direction of future research | Pages 7-8 |
| Conclusion | 11 | *Conclusions*  Summarise key conclusions, in a concise manner  Outline scope for and direction of future research |  |
| Additional information | 12a | *Registration*  In accordance with the Declaration of Helsinki*, state the unique research registration number and where it was registered, with a hyperlink to the registry entry (*this can be obtained from ResearchRegistry.com, ClinicalTrials.gov, ISRCTN etc.*)  *N.B. All retrospective studies should be registered before submission; it should be stated that the research was retrospectively registered.*  **‘Every research study involving human subjects must be registered in a publicly accessible database before recruitment of the first subject’* | Page 8 |
|  | 12b | *Ethical approval*  Whether ethical approval was needed or not, stated explicitly  Reason(s) why ethical approval was/was not needed  Name of the body giving ethical approval and approval number | Page 8 |
|  | 12c | *Informed consent*  State explicitly whether informed consent was obtained, or not.  State reason(s) why informed consent was/was not obtained  State the nature of consent (e.g. verbal, written, digital/virtual)*  The authors must provide evidence of consent, where applicable, and if requested by the journal Consent should be provided for both the original intervention/procedure and publication of the study  *If consent was not provided by the patient, explain why (e.g. death of the patient and consent provided by next of kin). If the patient or family members were untraceable, then document the tracing efforts undertaken | Page 8 |
|  | 12d | *Protocol*  Give details of protocol (a priori or otherwise) including how to access it (e.g. web address, DOI etc.)  Give details of protocol registration (e.g. protocol registration number, protocol registry's name etc.)  If published in a journal, cite and provide a full reference  If applicable, detail any amendments made to the original protocol, giving reasons why the changes were made | Not applicable |
| Declarations | 13a | *Contributorship*  Acknowledge any patient and/or public and/or professional involvement in research  Report the extent of involvement of each contributor, specifically stating what they contributed to (e.g. patient recruitment, defining research outcomes, dissemination of results etc.). | Page 8 |
|  | 13b | *Conflicts of interest*  Conflicts of interest, if any, are described | Page 9 |
|  | 13c | *Funding*  Sources of funding (e.g. grant details), if any, are clearly stated  Role of funder stated  Guarantor named | Page 8 |
|  | 13d | *Data sharing statement*  Explicitly state whether or not the datasets generated during study are available on request | Page 8 |

Supplementary Table 2: Summary of neoadjuvant treatment-related adverse events (neoTRAEs) and surgical complications according to ≥75% and <75% of neoadjuvant immunotherapy received before 14:00 h.

| **AEs/Surgical complications** | **Grade** | **≥75% group**  **(N = 123)** | **<75% group**  **(N = 45)** | ***P* value** |
| --- | --- | --- | --- | --- |
| **neoTRAEs** | Any grade | 63 (51.2%) | 17 (37.8%) | 0.122 |
|  | Grade 3 or 4 | 15 (12.2%) | 4 (8.9%) | 0.549 |
| Neutrophil count decreased | Any grade | 30 (24.4%) | 6 (13.3%) | 0.122 |
|  | Grade 3 or 4 | 13 (10.6%) | 3 (6.7%) | 0.445 |
| Alanine aminotransferase increased | Any grade | 14 (11.4%) | 4 (8.9%) | 0.644 |
|  | Grade 3 or 4 | 0 | 1 (2.2%) | 0.097 |
| Alopecia | Any grade | 12 (9.8%) | 4 (8.9%) | 0.865 |
|  | Grade 3 or 4 | 0 | 0 | - |
| Aspartate aminotransferase increased | Any grade | 10 (8.1%) | 1 (2.2%) | 0.170 |
|  | Grade 3 or 4 | 1 (0.8%) | 0 | 0.544 |
| Rash acneiform | Any grade | 6 (4.9%) | 2 (4.4%) | 0.907 |
|  | Grade 3 or 4 | 0 | 0 | - |
| Anemia | Any grade | 5 (4.1%) | 2 (4.4%) | 0.913 |
|  | Grade 3 or 4 | 0 | 0 | - |
| Flu like symptoms | Any grade | 3 (2.4%) | 1 (2.2%) | 0.935 |
|  | Grade 3 or 4 | 0 | 0 | - |
| Fever | Any grade | 3 (2.4%) | 1 (2.2%) | 0.935 |
|  | Grade 3 or 4 | 0 | 0 | - |
| Platelet count decreased | Any grade | 2 (1.6%) | 1 (2.2%) | 0.796 |
|  | Grade 3 or 4 | 0 | 0 | - |
| Palmar-plantar erythrodysesthesia syndrome | Any grade | 2 (1.6%) | 0 | 0.390 |
|  | Grade 3 or 4 | 0 | 0 | - |
| Diarrhea | Any grade | 2 (1.6%) | 0 | 0.390 |
|  | Grade 3 or 4 | 0 | 0 | - |
| Anorexia | Any grade | 2 (1.6%) | 0 | 0.390 |
|  | Grade 3 or 4 | 0 | 0 | - |
| Immune-related pneumonitis | Any grade | 2 (1.6%) | 1 (2.2%) | 0.796 |
|  | Grade 3 or 4 | 0 | 0 | - |
| Hypothyroidism | Any grade | 2 (1.6%) | 0 | 0.390 |
|  | Grade 3 or 4 | 0 | 0 | - |
| Creatinine increased | Any grade | 1 (0.8%) | 2 (4.4%) | 0.116 |
|  | Grade 3 or 4 | 0 | 0 | - |
| Hyperuricemia | Any grade | 1 (0.8%) | 0 | 0.544 |
|  | Grade 3 or 4 | 0 | 0 | - |
| Nausea | Any grade | 1 (0.8%) | 0 | 0.544 |
|  | Grade 3 or 4 | 0 | 0 | - |
| **Surgical complications** | Any grade | 30 (24.4%) | 8 (17.8%) | 0.364 |
|  | Grade 3 or 4 | 4 (3.3%) | 0 | 0.221 |
| Pneumonia | Any grade | 10 (8.1%) | 3 (6.7%) | 0.753 |
|  | Grade 3 or 4 | 1 (0.8%) | 0 | 0.544 |
| Blood transfusions | Any grade | 4 (3.3%) | 0 | 0.221 |
|  | Grade 3 or 4 | 0 | 0 | - |
| Prolonged air leak | Any grade | 4 (3.3%) | 2 (4.4%) | 0.712 |
|  | Grade 3 or 4 | 0 | 0 | - |
| Arrhythmia | Any grade | 4 (3.3%) | 1 (2.2%) | 0.728 |
|  | Grade 3 or 4 | 1 (0.8%) | 0 | 0.544 |
| Second thoracic drainage | Any grade | 2 (1.6%) | 1 (2.2%) | 0.796 |
|  | Grade 3 or 4 | 0 | 0 | - |
| Acute respiratory distress syndrome | Any grade | 1 (0.8%) | 0 | 0.544 |
|  | Grade 3 or 4 | 1 (0.8%) | 0 | 0.544 |
| Bronchopleural fistula | Any grade | 1 (0.8%) | 0 | 0.544 |
|  | Grade 3 or 4 | 1 (0.8%) | 0 | 0.544 |
| Chylothorax | Any grade | 1 (0.8%) | 1 (2.2%) | 0.456 |
|  | Grade 3 or 4 | 0 | 0 | - |

Supplementary Table 3: Surgical details according to ≥75% and <75% of neoadjuvant immunotherapy received before 14:00 h.

| **Variables** | **All**  **(N = 168)** | **≥75% group**  **(N = 123)** | **<75% group**  **(N = 45)** | ***P* value** |
| --- | --- | --- | --- | --- |
| **Incision approach, n (%)** |  |  |  |  |
| Video-assisted thoracoscopic surgery (VATS) | 86 (51.2%) | 65 (52.9%) | 21 (46.6%) | 0.344 |
| Robot-assisted thoracoscopic surgery (RATS) | 14 (8.3%) | 10 (8.1%) | 4 (8.9%) |  |
| Thoracotomy | 68 (40.5%) | 48 (39.0%) | 20 (44.5%) |  |
| **Extent of resection, n (%)** |  |  |  |  |
| Wedge resection | 2 (1.2%) | 2 (1.6%) | 0 | 0.290 |
| Segmentectomy | 1 (0.6%) | 1 (0.8%) | 0 |  |
| Lobectomy | 139 (82.7%) | 105 (85.3%) | 34 (75.5%) |  |
| Bilobectomy | 16 (9.5%) | 8 (6.5%) | 8 (17.8%) |  |
| Pneumonectomy | 10 (6.0%) | 7 (5.7%) | 3 (6.7%) |  |
| **Operative time, minute** | 194.5 (144.3-253.8)^*^ | 201.0 (143.0-266.0)^*^ | 192.8±73.2^#^ | 0.152 |
| **Estimated blood loss, ml** | 100.0 (20.0-187.5)^*^ | 100.0 (20.0-200.0)^*^ | 100.0 (20.0-175.0)^*^ | 0.513 |
| **Length of postoperative hospital stay, day** | 7.0 (6.0-10.0)^*^ | 7.0 (6.0-10.0)^*^ | 7.0 (6.0-9.0)^*^ | 0.538 |
| **Postoperative 30-day mortality, n (%)** | 0 | 0 | 0 | - |
| **Major pathologic response, n (%)** | 95 (56.5%) | 73 (59.3%) | 22 (48.9%) | 0.300 |
| **Pathologic complete response, n (%)** | 48 (28.6%) | 39 (31.7%) | 9 (20.0%) | 0.177 |

^*^median (interquartile range); ^#^mean±standard deviation.

Supplementary Table 4: Firth’s penalized Cox regression for variables associated with disease-free survival and overall survival in the original cohort.

| **Variables** | **Disease-free survival** | | **Overall survival** | |
| --- | --- | --- | --- | --- |
|  | **HR (95% CIs)** | ***P* value** | **HR (95% CIs)** | ***P* value** |
| **Age** |  |  |  |  |
| <60 years old | Reference |  | Reference |  |
| ≥60 years old | 1.420 (0.624-3.234) | 0.403 | 1.049 (0.235-4.678) | 0.950 |
| **Sex** |  |  |  |  |
| Male | Reference |  | Reference |  |
| Female | 3.128 (0.758-9.092) | 0.115 | 1.866 (0.215-6.189) | 0.572 |
| **Smoking history** |  |  |  |  |
| Current/former | Reference |  | Reference |  |
| Never | 0.723 (0.278-1.879) | 0.505 | 0.300 (0.072-1.250) | 0.098 |
| **Histology, n (%)** |  |  |  |  |
| Squamous cell carcinoma | Reference |  | Reference |  |
| Adenocarcinoma | 0.633 (0.229-1.748) | 0.378 | 1.713 (0.313-9.383) | 0.535 |
| LELC | 0.295 (0.056-1.545) | 0.148 | 0.015 (0.001-1.939) | 0.090 |
| Others | 1.551 (0.198-2.818) | 0.676 | 1.543 (0.067-5.432) | 0.786 |
| **PD-L1 expression** |  |  |  |  |
| Unknown | Reference |  | Reference |  |
| ≥1% | 2.153 (0.663-6.991) | 0.202 | 0.199 (0.012-3.215) | 0.256 |
| <1% | 2.322 (0.855-6.308) | 0.099 | 1.562 (0.298-8.183) | 0.598 |
| **Baseline tumour size** |  |  |  |  |
| <5 cm | Reference |  | Reference |  |
| ≥5 cm | 0.742 (0.342-1.612) | 0.451 | 2.282 (0.497-10.470) | 0.289 |
| **Neoadjuvant ICIs** |  |  |  |  |
| PD-1 inhibitor | Reference |  | Reference |  |
| PD-L1 inhibitor | 0.110 (0.007-1.659) | 0.111 | 0.126 (0.003-6.201) | 0.297 |
| **Neoadjuvant cycles** |  |  |  |  |
| 1-2 | Reference |  | Reference |  |
| ≥3 | 2.531 (1.026-6.242) | 0.044 | 1.633 (0.305-8.743) | 0.567 |
| **ToDA of neoIO** |  |  |  |  |
| ≥75% before 14:00 h | Reference |  | Reference |  |
| <75% before 14:00 h | 2.141 (0.923-4.969) | 0.076 | 3.295 (0.831-13.072) | 0.090 |
| **Mean timing interval** |  |  |  |  |
| ≥23.5 hours | Reference |  | Reference |  |
| <23.5 hours | 0.668 (0.239-1.868) | 0.441 | 0.063 (0.007-0.533) | 0.011 |
| **Radiographic response** |  |  |  |  |
| PR + CR | Reference |  | Reference |  |
| SD + PD | 0.928 (0.399-2.157) | 0.862 | 0.040 (0.004-0.411) | 0.007 |
| **Pathologic TNM staging** |  |  |  |  |
| p0 | Reference |  | Reference |  |
| pI | 1.504 (0.485-4.664) | 0.479 | 4.628 (0.213-10.385) | 0.329 |
| pII | 2.008 (0.492-8.193) | 0.331 | 2.327 (0.073-7.661) | 0.633 |
| pIII | 8.869 (3.982-19.412) | <0.001 | 6.124 (1.970-9.084) | 0.021 |
| **Major pathologic response** |  |  |  |  |
| No | Reference |  | Reference |  |
| Yes | 1.006 (0.364-2.778) | 0.991 | 0.371 (0.063-2.201) | 0.275 |
| **Extent of resection** |  |  |  |  |
| Sublobar resection | Reference |  | Reference |  |
| Lobectomy | 0.139 (0.027-0.715) | 0.018 | 0.501 (0.010-4.801) | 0.728 |
| Bilobectomy | 0.096 (0.011-0.843) | 0.035 | 0.490 (0.004-6.513) | 0.776 |
| Pneumonectomy | 0.248 (0.022-2.803) | 0.260 | 0.886 (0.006-7.327) | 0.962 |
| **Adjuvant therapy** |  |  |  |  |
| Yes | Reference |  | Reference |  |
| No | 0.585 (0.155-2.203) | 0.428 | 0.070 (0.007-0.697) | 0.023 |

HR, hazard ratio; CIs, confidence intervals; LELC, lymphoepithelioma-like carcinoma; PD-L1, programmed death ligand 1; ICIs, immune checkpoint inhibitors; ToDA, time-of-day; neoIO, neoadjuvant immunotherapy; PD-1, programmed death 1; PR, partial response; CR, complete response; SD, stable disease; PD, progressive disease.

Supplementary Figure 1: The unadjusted and inverse probability of treatment weighting-adjusted standardized mean differences between the ≥75% and <75% of neoadjuvant immunotherapy received before 14:00 h. PD-L1, programmed death ligand 1; PD-1, programmed death 1.
